# Idiopathic Thrombocytopenic Purpura: Current Limitations and Management

**DOI:** 10.7759/cureus.49313

**Published:** 2023-11-23

**Authors:** Rakshanda Thakre, Pankaj Gharde, Mohit Raghuwanshi

**Affiliations:** 1 Surgery, Jawaharlal Nehru Medical College, Datta Meghe Institute of Higher Education and Research, Wardha, IND; 2 Community Medicine, Jawaharlal Nehru Medical College, Datta Meghe Institute of Higher Education and Research, Wardha, IND

**Keywords:** immune thrombocytopenic purpura, autoimmune disorder, thrombocytopenia, hemorrhage, bleeding disorder

## Abstract

Idiopathic thrombocytopenic purpura (ITP), also known as immune thrombocytopenia, is a blood disorder characterized by a reduction in the number of platelets. A reduction in the number of platelets beyond the normal levels leads to several consequences. A severe reduction in blood platelet levels leads to a rash of purple spots on the skin, joints, etc. due to leakage in the small blood vessels, easy bruising, bleeding gums, intestinal bleeding, and hemorrhage. Suppose a case of ITP resolves in fewer than six months. In that case, it is an acute case of ITP. Still, if a case settles in more than six months, it is a case of ITP. The cause of a reduced platelet count can be increased peripheral destruction or impaired production; this is termed an autoimmune condition in which the body's immune system attacks platelets thinking it to be a foreign antigen. ITP in children occurs commonly following a previous viral attack. Even though evaluating patients' reports is useful for understanding and guiding the treatment, these estimates might not be regularly evaluated in clinical settings. First-line drugs in the treatment of ITP are corticosteroids, and long-term use of these drugs has several side effects, such as excessive increase in weight, mental health disturbances, and sleep disturbances; additional therapies to treat hemorrhage are usually momentary. As a result, it is essential to recognize the flaws in current procedures and adopt innovative measures for the management and minimization of difficulties.

## Introduction and background

Immune thrombocytopenic purpura

Idiopathic thrombocytopenic purpura (ITP) is a remote condition of thrombocytopenia in which the bone marrow does not show any change, and there is an absence of any other cause of thrombocytopenia. The decrease in platelets is due to impaired production or peripheral destruction. The cause of destruction in most patients is due to the presence of antiplatelet membrane glycoprotein antibodies. The cases of primary ITP are isolated, i.e., without any predisposing or underlying cause, but the cases of secondary ITP are secondary to autoimmune diseases like systemic lupus erythematosus, viral infections generally in children, lymphoproliferative disorders, neoplasms, etc. [1-3]. The cases of ITP in children are mostly secondary to febrile conditions, usually posted viral attacks. ITP has been attributed to production of platelets that express viral antigens, generation of antiviral antibodies that have cross-reactivity with viral antigens, formation of antibodies by epitope spread, or immune binding complexes [4]. Megakaryocytes are precursors of platelets; they attack the spread of intravascular coagulation, syndromes of microangiopathies, and hemophagocytosis, which leads to alteration of platelet surface and hence hastens the clearance [5].

The currentlimitations in ITP cases

The patient experiences fatigue due to decreased platelets, which the patient often ignores. Fatigue is one of the major shortcomings of patient-reported outcomes in ITP. The main focus of the physicians is to manage the bleeding, because of which other side effects like how the patient feels and functions, anxiety, fear, frustration, etc. are ignored. Fatigue is one of the important components of health-related quality of life (HRQOL), which is an individual's or a group's mental and physical health over time [6]. A persistent chronic case of thrombocytopenia presents with severe bleeding, which requires cessation with the help of prompt treatment; the treatment varies depending on the progress the patient makes. The improvement in platelet count also helps in improving HRQoL. Even after medication, fatigue does not improve even if the platelet count is raised. There is little data to support the idea that starting treatment early in adult ITP patients would stop the progression of chronic illness [6]. Fatigue in ITP patients is mainly because of excessive bleeding, and prolonged bleeding due to chronic disease causes iron deficiency and anemia. This deficiency of iron and anemia is thought to be the main cause of fatigue in these patients. ITP is a disorder in which the platelet count reduces; platelet is a component of blood that prevents bleeding. Giving corticosteroids, immunoglobulin, and anti-D improves the platelet count but cannot compensate for the blood loss immediately; eventually, as the platelet count increases after treatment and blood volume and stores of iron and red blood cells replenish, the fatigue improves gradually. Improvement in fatigue can be assessed using HRQoL and Patient Assessment Questionnaire (PAQ). According to studies, transdiagnostic factors account for a significantly larger percentage of fatigue in ITP than does disease activity. A large number of these variables, such as physical activity, vitamin D, and overall health and functionality, are potentially adjustable. As a result, individuals could make suitable candidates for therapies like cognitive behavioral therapy, exercise therapy, self-management programs, vitamin D supplementation, and/or sleep interventions, and all these factors over time can help improve fatigue [7,8]. The baseline severity score helps predict the future risk of fatigue and cognitive dysfunction. Fatigue severity is not related to disease severity in ITP patients, as fatigue intensity remains stable over a period of time. Treatments that aim at reducing autonomic impairment and hence may minimize fatigue severity and cognitive impairment can make potential contributions toward improving quality of life [9]. ITP PAQ is a questionnaire consisting of six scales: physical health (including fatigue/sleep), emotional health, social activity, work, women's reproductive health, and overall quality of life. These scales are monitored, and they also help to evaluate HRQoL. The PAQ was made to analyze HRQoL and the intensity of the disease. The scale has the exception that it does not include the reproductive health scale [10]. Another tool used for the measurement of HRQoL in children (specifically between the ages of 2 and 18 years) with ITP is The Kids ITP Tool (KIT) [11]. There is no specific gold standard to confirm ITP; ITP is an exclusionary diagnosis. Diagnosis can be approved based on the response to treatment, which is also not absolute. Corticosteroids remain the first line of drugs for the cure of ITP. However, due to their mechanism of action, newer drugs are now widely accepted, namely rapamycin, fostamatinib, FcRn, and BTK (Bruton tyrosine kinase) inhibitors. The cases of refractory ITP are the cases of ITP that do not show any response to treatment (for example, there is no response to treatment with rituximab and TPO [thrombopoietin] agents) [12]. Intravenous Immunoglobulin is also given in the cure of ITP. Research has linked the use of intravenous immunoglobulin to treat ITP to an enhanced danger of acute myocardial infarction (troponin I is a marker specific for cardiac muscles and increases 6-8 hours after myocardial injury) [13].

## Review

Methodology

**Figure 1:**
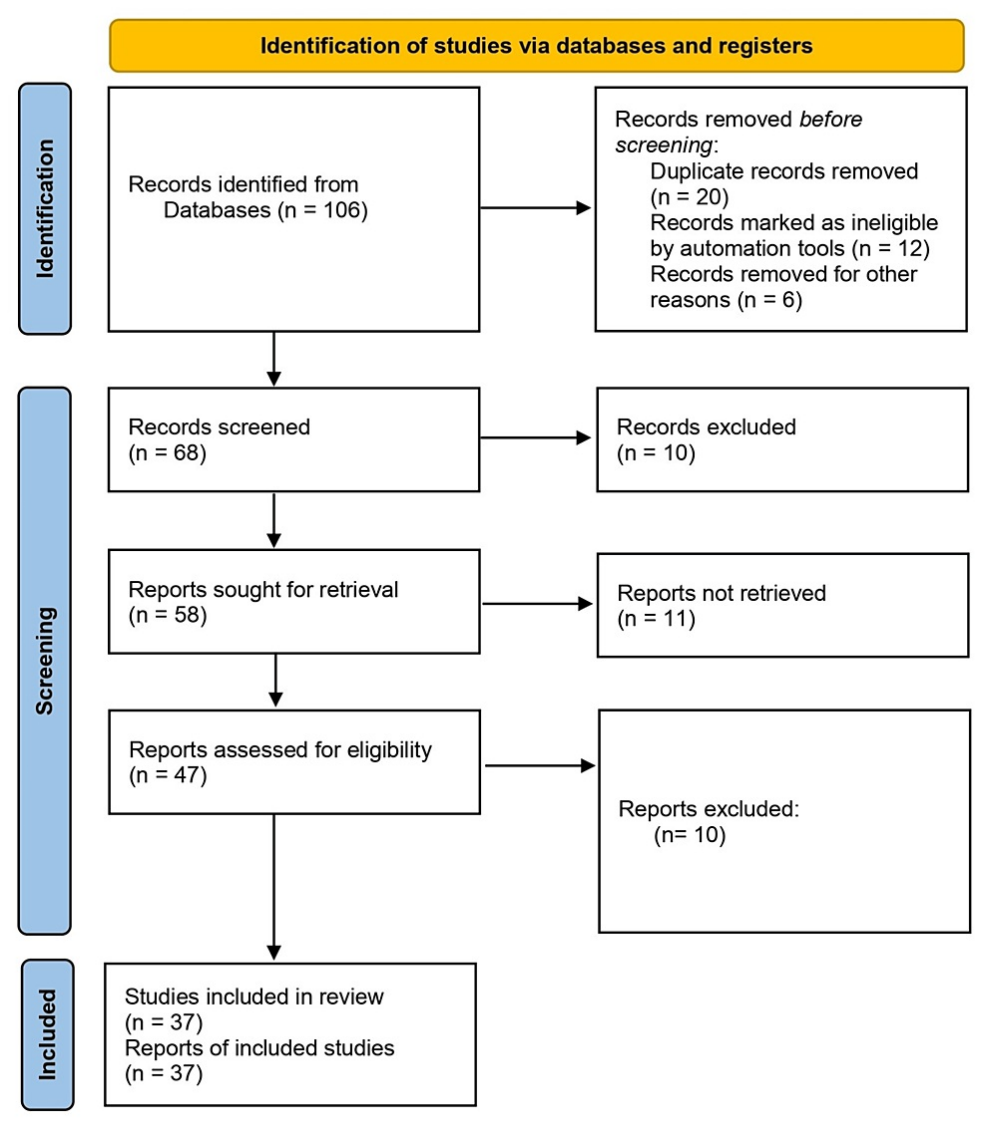
PRISMA flow chart of the included studies in the review PRISMA: Preferred Reporting Items for Systematic Reviews and Meta-Analyses.

To ensure a thorough and up-to-date examination of recent developments, current limitations, and management of patients with ITP, a meticulous search methodology was implemented. Several relevant articles from multiple databases were studied, which include PubMed. The search spanned articles from the years 1986-2023 and these articles were analyzed. For a precise search process, a variety of keywords, headings, molecular diagnoses, and subheadings relevant to ITP, its management, current limitations, pathophysiology, and aspects related to ITP were analyzed and employed. This adaptable strategy was tailored to the specific indexing conventions of each database to optimize search sensitivity. Inclusion criteria focused on high-quality evidence, original research, systematic review, and clinical trials. Conversely, exclusion criteria were applied to rule out studies with insufficient data, outdated information, or non-English language publications. The screening process involved a review of titles and abstracts for initial studies, followed by a thorough examination of the full text of the article. The final review includes relevant information from a curated selection of articles, totaling 37 articles, providing information about ITP, its pathophysiology, recent development, current limitations, and management. This search methodology (Figure 1) endeavors to transparently outline the rigorous steps taken to identify, assess, and include relevant literature in this comprehensive review.

Pathogenesis

As mentioned above, ITP is a condition in which impaired platelet production or increased platelet destruction leads to complications like bleeding. If the prognosis of the disorder is based on a decrease in the production of platelets, then the risk of the illness recurrence rises. Still, if the mechanism is based on platelet peripheral destruction, then the response to medications is good, and the risk of relapse is low [14]. Secondary ITP - It is thrombocytopenia secondary to any diseased condition, which is usually a viral attack. A few of the conditions that cause ITP are measles-mumps-rubella vaccine (ITP-MMR), *Helicobacter pylori* (ITP-HP), cytomegalovirus (ITP-CMV), varicella zoster virus (ITP-VZV), hepatitis C virus (ITP-HCV), human immunodeficiency virus (ITP-HIV), etc. These are explained in Table 1. ITP-MMR is an acute attack of ITP occurring after vaccination. Thrombocytopenia develops usually after 42 days of exposure. This type of thrombocytopenia is responsive to corticosteroids in most cases. ITP-HP is an investigation and diagnosis of a case of *Helicobacter pylori* that can be confirmed with the help of a C-urea breath test and microscopic investigation of stool samples for bacterial or gastric biopsy. ITP-HP is a long-lasting platelet response to bacterial eradication. Geographic variation has been observed in cagA virulence genes in HP strains. In these cases, the platelets cross-react with the cagA genes. ITP-CMV, in this type of cytomegalovirus, delays the recovery of platelets after the transplantation of bone marrow and causes congenital thrombocytopenia. Few cases show recovery after treatment against CMV in fourth refractory patients. ITP-VZV can affect neonates through vertical transmission. At first, there is exanthema and then occurs bleeding (hemorrhage) mainly at extracutaneous sites. Few cases show reduced survival of platelets in the unaffected bone marrow. ITP-HCV is thrombocytopenia in relation to hepatitis, which has a multifactorial etiology and is poorly understood. Polyclonal B cells are activated by structural protein E2 by binding to CD81. As the disease advances, there is impaired production of TPO and impaired production of platelets is due to infection of the megakaryocytes. In ITP-HIV, the main mechanism is rapid clearance and impaired production of platelets. Antibodies present in sera cross-react with an amino acid of GPIIIa (Glycoprotein IIIa). NADPH (nicotinamide adenine dinucleotide phosphate hydrogen) is activated by the fragmentation of platelets which is carried out by anti-GPIIIb/IIIa, and there is the release of peroxide and other oxygen species (dexamethasone inhibits the fragmentation of platelets) [15].

**Table 1:** ITP that occurs after exposure to viruses. ITP, immune thrombocytopenic purpura.

S. no.	Types of ITP	Organism involved	Causes	Special feature about investigation/diagnosis/treatment
1.	ITP-MMR	Measles–mumps-rubella (MMR) vaccine	Acute attack of ITP occurring after MMR vaccination. Thrombocytopenia develops usually after 42 days of exposure.	This type of thrombocytopenia is responsive to corticosteroids in most of the cases.
2.	ITP-HP	*Helicobacter pylori* (*H. pylori*)	Infection with bacteria *H. pylori* is the main cause of ITP-HP. ITP-HP is a long-lasting platelet response to bacterial eradication. Geographic variation in cag A virulence genes in HP strains. In these cases, the platelets cross-react with the cag A genes.	Investigation and diagnosis of a case of *H. pylori* can be confirmed with the help of a C-urea breath test, and microscopic investigation of stool samples for bacterial or gastric biopsy.
3.	ITP-CMV	Cytomegalovirus	It is caused by viral infection with cytomegalovirus. The cytomegalovirus delays the recovery of platelets after the transplantation of bone marrow and causes congenital thrombocytopenia.	Few cases show recovery after treatment against CMV in fourth refractory patients.
4.	ITP-VZV	Varicella zoster virus	Can affect neonates through vertical transmission. At first, there is exanthema, and then occurs bleeding (hemorrhage) mainly at extracutaneous sites.	Few cases show reduced survival of platelets in the unaffected bone marrow. Few show response to acyclovir and IVIG (intravenous immunoglobulin) against the viral infection.
5.	ITP-HCV	Hepatitis C virus	Thrombocytopenia in relation to hepatitis has multifactorial etiology and is poorly understood. Polyclonal B cells are activated by structural protein E2 by binding to CD81.	As the disease advances, there is impaired production of thrombopoietin and impaired production of platelets is due to infection of the megakaryocytes. Direct-acting antiviral (DAA) tablets can be given against the virus.
6.	ITP-HIV	Human immunodeficiency virus	The main mechanism in these cases is rapid clearance and impaired production of platelets. Antibodies present in sera cross-react with an amino acid of GPIIIa. NADPH is activated by the fragmentation of platelets which is carried out by anti-GPIIIb/IIIa, and there is the release of peroxide and other oxygen species (dexamethasone inhibits the fragmentation of platelets).	Antiretroviral drugs are given with platelets.

Primary ITP 

In adult ITP, antiplatelet glycoprotein autoantibodies are sensitized by the activation of the RE system, i.e., the reticuloendothelial system, which causes the destruction of the platelets. The platelets are opsonized by autoantibodies and phagocytosed. Usually, the disease is chronic and organ-specific autoimmune hemorrhagic [16]. One of the major components of pathophysiology is dysfunctional cellular immunity. In the last 20 years, one of the important developments in the treatment of ITP is the use of IVIG and anti-D preparation [17]. GP IIb-IIIa (membrane glycoprotein) is present on the plasma membrane of the platelet. Upon platelet activation, they undergo conformational changes and adhere to each other. Membrane GP IIb-IIIa (integrin/CD41/CD61) is one of the major targets of antiplatelet antibodies. These are heterodimeric receptors for fibrinogen and are calcium-dependent. CD4+ T cells have helper activity and these help in the production of anti-GP IIb-IIIa antibodies. Platelet-reactive CD4+ T cells recognize GP IIb-IIIa as a major target antigen. Anti-GP IIb-IIIa bind to normal platelets. In ITP patients, these autoreactive T cells are responsible for producing antiplatelet antibodies. These autoreactive T cells recognize cryptic epitopes on GP IIb-IIIa. Cryptic epitopes are sequestered (hidden) antigenic determinants and are a part of antigens recognized by an antibody that is processed and presented more efficiently after an inflammatory response. GP IIb-IIIa does not produce cryptic epitopes by normal processing pathways [18-20]. Predisposing genetic factors are anti-inflammatory cytokines, transcription factors, chemokines, and major histocompatibility complex. Macrophages trigger an adaptive immune response in their capacity as antigen-presenting cells and destroy platelets in their capacity as effector cells. A study was done to see B cell (B lymphocyte) involvement, and it was observed that intravenous infusion of serum of patient affected with ITP into a normal patient's serum triggered thrombocytopenia. The humoral factor associated with ITP was IgG [21].

Limitations in current ITP cases

The limitation of patient-reported outcomes in ITP shows that physicians put more stress on treating the low platelet count, and the treatment of fatal bleeding is often ignored. How the patient feels and functions is majorly affected. In HRQoL, fatigue is one of the major elements and is a consequence of ITP; it makes patients weak and infirm. Symptoms of fatigue are often ignored because it is a common complaint of a healthy as well as a chronically ill patient. The extent of fatigue cannot be monitored easily and accurately as there are limited studies and there is limited data that is available to measurement of the extent of fatigue, and there is an unknown pathophysiology of the underlying cause of fatigue. The patient also feels anxiety and frustration following the disease [9,22]. Several instruments are used in the assessment of ITP, including PAQ, which contains 44 questions and takes roughly 10-15 minutes to solve. It consists of six scales, namely physical health (including fatigue/sleep), emotional health, social activity, work, women's reproductive health, and overall quality of life. These scales are monitored, and they also help to evaluate HRQoL. Another measure used is KIT. It is used for children and contains three questionnaires: one is for assessment of children of age group 7-17 years; the other is for age group 2-17 years, and one for measuring the complete impact of disease on a patient's life; it is also used to assess HRQoL [10,23-26].

Limitations of Current Therapies for Patients With Newly Diagnosed ITP

Some therapies are required to treat bleeding, improve platelet count, cause cessation of bleeding, improve HRQoL, decrease fear and anxiety, and decrease bleeding. Even after treatment, it is seen that the fatigue of the patient with ITP has not improved. There is no clear evidence that in new cases of ITP use, treatment prevents the development of chronic diseases [27-29]. 

Pregnancy and thrombocytopenia

There are high chances of thrombocytopenia in late pregnancy. Those who don't have pregnancy-related causes for low platelet count must be evaluated for other underlying conditions for thrombocytopenia. Pregnancy-specific causes for thrombocytopenia may include pre-eclampsia and its severe form of hemolysis with elevated liver enzymes and low platelets (HELLP). Treatment and management of ITP in pregnant females are challenging as the first-line drugs used, like corticosteroids and immunoglobulins, can cause several side effects as they can induce negative effects on fetal intrauterine growth (IUGR i.e intrauterine growth retardation), neonatal birth weight, excessive weight gain in the pregnant female, suppressed immunity, etc. The neonatal thrombocytopenia risk must be taken into account [30].

Pediatric ITP 

It is usually self-limiting unlike adult ITP; adult ITP might progress to a chronic case of ITP [31]. Out of every 100,000 children, 5-10 children are affected by ITP every year. Female predominance is seen in cases of ITP (2:1 ratio). The risk and incidence of autoimmune diseases specifically systemic autoimmune diseases occur more commonly in females than in males; hence, the predominance of females is greater than males in ITP disorders [31,32]. The difference between pediatric and adult ITP as mentioned in Table 2 lies in presenting platelet count, family history of thrombocytopenia, and rates of treatment. In pediatric ITP the presenting platelet count is 18.1 × 10^9^/L, whereas in adult ITP the count is 25.4 × 109/L. Family history of thrombocytopenia is more significant in adult ITP as compared to pediatric ITP. Rates of treatment are more in pediatric ITP as they respond to treatment better than adults with ITP [32,33]. 

**Table 2:** Comparison of adult and pediatric ITP. ITP, immune thrombocytopenic purpura.

Heading	Pediatric ITP	Adult ITP
Presenting platelet count	18.1 × 10^9^/L	25.4 × 10^9^/L
Family history of thrombocytopenias	2%	3%
Rates of treatment	80%	71%

Management and treatment 

We advise either prednisone (0.5-2.0 mg/kg/day) or dexamethasone (40 mg/day for four days) as the initial corticosteroid therapy is prescribed to an adult suffering from ITP. Dexamethasone may be preferable over prednisone if the speed of the platelet count response is emphasized, as the reaction at seven days was better with dexamethasone [34,35]. In patients with symptomatic ITP, anti-D Immunoglobulin can be a useful alternative treatment for elevating platelet counts. Anti-RhD immunoglobulin (anti-D) is widely used in treating acute and chronic ITP. FDA (Food and Drug Administration)-approved dose of 50 mg/kg increases platelet count in approximately 80% of pediatric ITP cases and 70% of adult ITP cases. A rapid elevation in platelet number without a reduction in hemoglobin is seen with a higher dose of 75 micrograms per kg. It is observed that patients with failed splenectomy anti-D prove to be ineffective [36]. Adult immune thrombocytopenia is typically chronic. There are lots of patients who need various therapeutic approaches. One of the few non-immunosuppressive treatments that can quickly raise platelet counts in individuals with severe thrombocytopenia is anti-D therapy combined with IVIG. Most ITP patients may have an increase in circulating platelets due to a reversible interaction of sensitized RBC with phagocytic cells and a low-grade killing of these cells rather than an Fc receptor blockage involved in platelet sequestration [36,37]. Steroids, often known as corticosteroids, work to stop bleeding by lowering the generation of antibodies that attack platelets. Within two to four weeks of beginning steroids, the platelet count will increase if they are successful. Side effects can include mood swings, acne, weight gain, irritability, stomach irritation, and problems sleeping.

Management in adult

Inpatient Management Versus Outpatient Management

There can be two types of cases for management of ITP, if it is a newly diagnosed case of ITP having a platelet count of <20x10^9^/L and is asymptomatic or presents with mild mucocutaneous bleeding and another being platelet count of ≥20x10^9^/L and is asymptomatic with mucocutaneous bleeding. It is recommended that a six-week course of prednisone is favored over a short course. Corticosteroids used for initial therapy are prednisone 0.5-2.0 mg/kg/day or dexamethasone 40 mg/day for four days. Second-line therapies in adults having chronic ITP for more than three months who have developed resistance to corticosteroids and hence are nonresponsive to corticosteroids or are corticosteroid-dependent second-line drugs like TPO receptor agonists (eltrombopag or romiplostim); rituximab can be used for management and treatment. Splenectomy can be done in severe cases. Before splenectomy physician must ensure that the patient is fully immunized, and the patient must be made aware of the fever that follows post-splenectomy and the management of the same. Patients under corticosteroid treatment should be monitored for potential adverse effects. The side effects of corticosteroids include hypertension, hyperglycemia, sleep and mood disturbances, gastric irritation or ulcer formation, glaucoma, myopathy, and osteoporosis. In first-line therapies in children, in pediatric non-life-threatening bleeding, corticosteroids are preferred over IVIG, or anti-D immunoglobulin is used when corticosteroids are contraindicated. Children with newly diagnosed cases of ITP underwent a randomized control experiment to evaluate IVIG with anti-d immunoglobulin. Patients with a platelet count less than 20×10^9^/L were given IVIG 2 g/kg and anti-D Ig 75 μg/kg and 50 μg/kg; it was observed that there was an increase in platelet count remarkably among IVIG groups than anti-D Ig patients. The study suggested that IVIG is better tolerated and more effective than anti-D Ig. Further studies are required to confirm this finding and compare IVIG and anti-D Ig to be used in treating ITP [38].

Ayurvedic Treatment for ITP

Indian Ayurveda classifies ITP under Tiryaka Raktapitta, a blood disorder. The target of the ayurvedic treatment is to give basic purification, and purgation to provide nourishment to the spleen and other organs. Later in treatment, oral medications and lifestyle modifications are done for 2-3 years and patients are monitored throughout this period, and the duration of the treatment may vary depending on the patient. The steroid medication is not stopped completely but the dose is reduced gradually. Snehapana and Virechana are taken, and the only adverse effects are temporary lack of appetite, loose motion, weariness, and vomiting. Ayurvedic medications with anticoagulant and antitumor properties, like Durwa, Amlaki, Shatavari, Ashwagandha, and Gokshura, aid in the patient's recuperation from ITP. Until and unless they are taken incorrectly, ayurvedic medications for the treatment of ITP are entirely natural and do not have any significant negative effects in the long term [39].

Allopathic Treatment

Steroids reduce the rate of platelet destruction and prevent bleeding, but have several side effects like stomach irritation, weight gain, high blood pressure, and acne whereas long-term side effects may include liver failure, heart attack, etc. IVIG is a protein containing antibodies that also slows the destruction of platelets; its side effects include flushing, headache, malaise, fever, chills, fatigue, and lethargy whereas its serious side effects include renal impairment, thrombosis, arrhythmia, aseptic meningitis, hemolytic anemia, and transfusion-related acute lung injury. 

The summary of the article is mentioned in Table 3. The table gives information about the findings of each study [1-39].

**Table 3:** Summary table of studies included in the review. ITP, idiopathic thrombocytopenic purpura; HELLP, hemolysis, elevated liver enzymes, and low platelets.

Authors	Year	Findings
Onisâi M et al., [1]	2019	An autoimmune hematological condition known as immune thrombocytopenia reduces platelets, which leads to bleeding. Although there are several therapeutic options, the likelihood of relapse and complications is significantly high.
Onisâi M et al., [2]	2012	Thrombocytopenia in pregnancy is majorly associated with preeclampsia and HELLP syndrome causing perinatal morbidity, prematurity, low birth weight, preterm delivery, etc. Therefore, close monitoring is required to recognize the cause and provide the best interventions.
Cortelazzo S et al., [3]	1991	Elderly patients affected with idiopathic thrombocytopenic purpura for a long duration of time have increased chances of severe hemorrhage.
Cines DB and Blanchette VS, [4]	2002	Pathophysiology of generation of antibodies against platelet glycoprotein IIb/IIIa complex and treatment modality for children and adult age group.
Neunert CE et al., [5]	2008	In spite of several new treatment options available, an appropriately powered therapeutic study is challenging to design to prevent bleeding at least during the first four weeks of diagnosis. Further future studies are required.
Terrell DR et al., [6]	2020	It would be easier for patients and doctors to successfully monitor a patient's health outside of just treating the laboratory results and outward signs of ITP. There should be future research on the care of newly diagnosed and refractory ITP cases with an emphasis on the current limitations of existing medicines.
van Dijk WEM et al., [8]	2022	Measures to decrease the incidences of fatigue in patients of chronic ITP.
Mitchell E et al., [9]	2019	Studies show that fatigue and cognitive impairment (CI) are common in ITP and the use of baseline scores to predict future severity could help in the treatment of ITP.
Mathias SD et al., [10]	2007	Development of ITP-Patient Assessment Questionnaire (ITP-PAQ) to assess health-related quality of life (HRQoL).
Klaassen RJ et al., [11]	2013	Kids ITP tools (KIT) is a measure to assess health-related quality of life (HRQoL) in children with ITP.
Miltiadous O et al., [12]	2020	Difficulty in diagnosis and treatment of refractory ITP, limitations in treatment, and role of combination treatment.
Paolini R et al., [13]	2000	Factors associated with a high risk of hemorrhagic complications include people with age more than 60 years.
Kistangari G and McCrae KR, [14]	2013	ITP caused due to secondary causes, its epidemiology and treatment.
Cines DB et al., [15]	2009	ITP caused due to secondary factors like immune deficiencies, infections, and environmental and genetic factors.
Panitsas FP et al., [16]	2004	Type 1 polarized immune response in chronic adult ITP.
Semple JW, [17]	2002	Pathogenesis of ITP by cell-mediated response and cytokine abnormality within adult ITP and mechanism of action of anti-D.
Kuwana M et al., [18]	2001	The immunodominant epitopes that are pathogenic CD4+ T cells in individuals with ITP are located in the amino-terminal regions of both GPIIb and GPIIIa.
Kuwana M, et al., [19]	1998	The pathogenic processes involved in the production of anti-platelet autoantibody include CD4+ and HLA-DR-restricted T cells to GP IIb/IIIa.
Wucherpfennig KW and Strominger JL, [20]	1995	An important factor for understanding the pathogenesis of autoimmunity is molecular mimicry. A single T cell receptor can identify distinct but structurally similar peptides for multiple pathogens.
Audia S et al., [21]	2017	The pathophysiology of thrombocytopenia is described with the role of immune cells and treatment.
Cooper N et al., [22]	2021	World Impact Survey (iWISH) regarding the overall severity of ITP symptoms, patients and doctors generally agree, albeit there are some exceptions, such as fatigue.
Kirsch M et al., [23]	2013	Using and understanding the importance of patient-reported outcomes in patients with ITP.
Klaassen RJ et al., [24]	2007	Kids ITP tools and about newer modalities of health-related quality of life in children with ITP.
Kuter DJ et al., [25]	2010	Treatment of ITP patients with romiplostim, its advantages and disadvantages.
Kuter DJ et al., [26]	2008	Short-term and long-term effects of administration of romiplostim in splenectomized and non-splenectomized patients.
Adelborg K et al., [27]	2019	Cardiovascular events in patients with ITP.
Bruin M et al., [28]	2004	FCGR2B genotype, infections as a risk factor for the development of ITP in childhood.
Heitink-Pollé KM et al., [29]	2014	Clinical investigations of chronic ITP and prediction of cases of chronic ITP.
Eslick R and McLintock C, [30]	2020	ITP in pregnancy and its treatment modalities.
Despotovic JM et al., [31]	2018	Correlation of age with incidences of ITP, the chance of ITP increases with increasing age, especially after 60 years of age patients with ITP have high chances of hemorrhage.
Schifferli A et al., [32]	2018	Studies show more chances of bleeding in adults with ITP as compared to children with ITP.
Kühne T et al., [33]	2011	In children and adults with ITP, there is a difference in co-morbidities, diagnostic procedures, and treatment.
Saeidi S et al., [34]	2014	The chances of acute ITP are more in children as compared to adults, adults have increased chances of chronic ITP.
Park YH et al., [35]	2022	Decision regarding first-line and second-line treatment of ITP, showing studies carried out in Korea.
Cheung E et al., [36]	2009	Role of anti-RhD immunoglobulin in management of patients with ITP.
Salama A et al., [37]	1986	Role of IgG anti-RhD in management of patients with chronic ITP.
Alioglu B et al., [38]	2013	Studies show IVIG is a better first-line treatment modality for patients with ITP.

## Conclusions

ITP is a rare disorder, and it is more frequent in females than males. The exact mechanism behind the impaired production and increased destruction remains unknown, though thought to be because of autoantibodies. Most of the cases occur after viral attacks, mainly in children. The treatment is majorly corticosteroid-based but has several side effects; alternative therapies in IVIG and anti-D immunoglobulin are available but are less effective and less acceptable. More studies need to be conducted to confirm the best treatment for ITP if corticosteroids are contraindicated. Blood reports must be evaluated and monitored constantly to confirm the diagnosis of ITP. A bone marrow aspiration test is also performed to examine platelet production; this test is not always mandatory and should be avoided in children and mild cases. The cause of the generation of autoantibodies remains unknown in primary or idiopathic ITP. Apart from corticosteroids which are other best treatments that can be used as first-line treatment, this remains unknown and requires more studies and investigations.
